# DNA sequences and distinct mechanisms for *ura4-595* and *ura4-294* alleles of *S. pombe*

**DOI:** 10.17912/micropub.biology.001139

**Published:** 2024-02-16

**Authors:** Reine U Protacio, Emory G Malone, Wayne P Wahls

**Affiliations:** 1 Biochemistry and Molecular Biology, University of Arkansas for Medical Sciences, Little Rock, Arkansas, United States

## Abstract

The *ura4* gene of the fission yeast *Schizosaccharomyces pombe* supports both positive and negative selection; consequently, this gene is widely employed as a powerful tool to study diverse biological processes. Here we report the DNA sequences of two functionally null alleles, *ura4-595* and *ura4-294* . The *ura4-595* allele has a four bp duplication of bp +63 to +66 (5’-CAAG-3’) within the ORF and the *ura4-294* allele has a nonsynonymous substitution (G to A) at bp +679. We infer that these alleles arose, respectively, by DNA polymerase template slipping and by nucleotide misincorporation (likely via cytosine deamination).

**Figure 1. DNA sequences and functionality of ura4 alleles.**
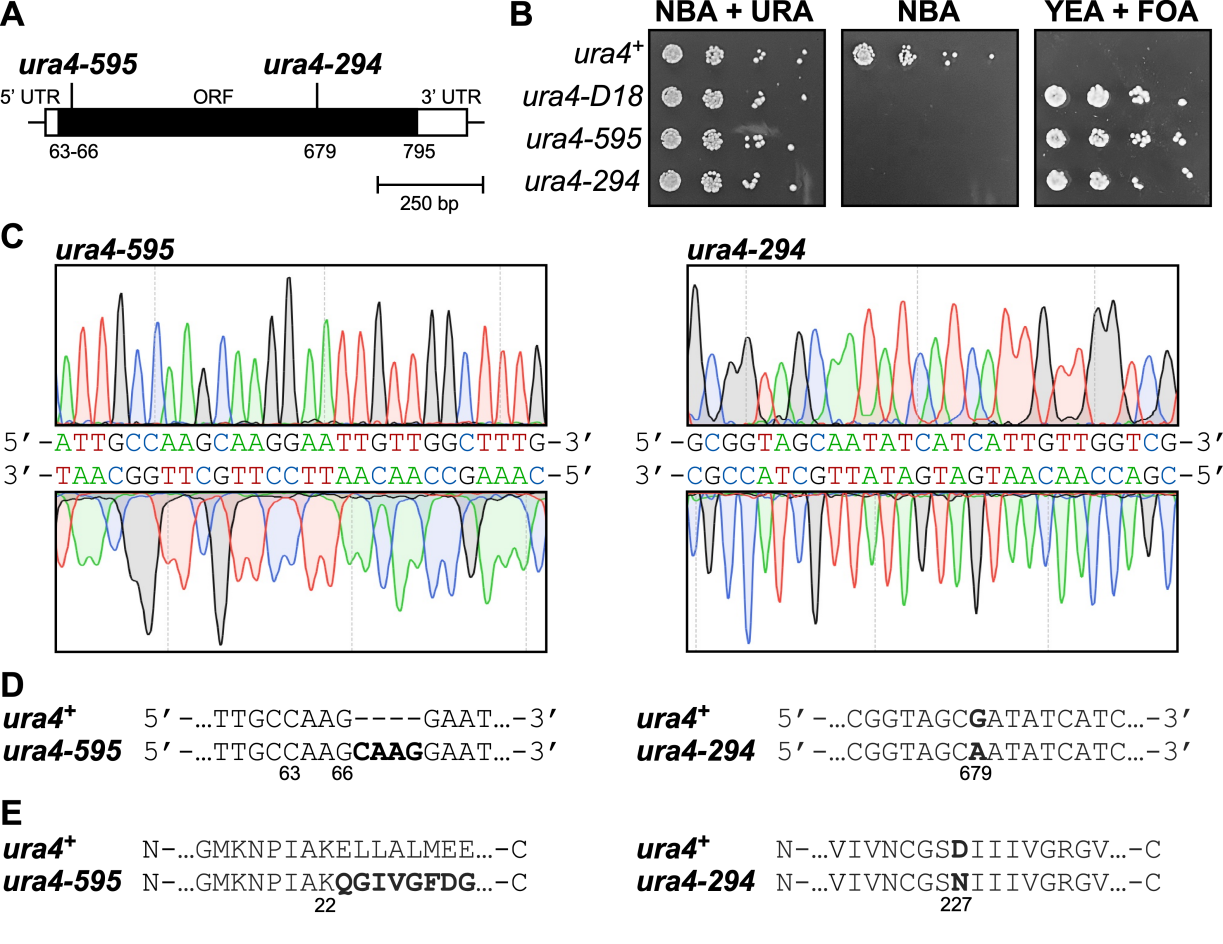
**A.** Schematic diagram of the *ura4* gene and the positions of alleles defined in this study. The *ura4* ORF is 795 bp in length and coordinates are numbered relative to the first nucleotide of the start codon (+1). **B.** Phenotypes of cells with *ura4-595* and *ura4-294* alleles. Serial dilutions of cells were plated on minimal media (NBA) that contains or lacks uracil (URA) and onto rich media that contains FOA (YEA + FOA). Cells expressing a wild-type Ura4 protein ( *ura4 ^+^* ) and null mutant cells ( *ura4-D18* ) that lack Ura4 protein provide controls. **C.** DNA sequences of the *ura4-595* and the *ura4-294* alleles. The chromatograms show the relevant sequences of each DNA strand. **D.** Alignments compare the DNA sequences of the mutant alleles to that of wild-type *ura4 ^+^* . A four bp duplication in *ura4-595* and a single bp substitution in *ura4-294* are highlighted (bold). **E.** Amino acid sequence changes (bold) encoded by alleles. The frameshift caused by *ura4-595* scrambles 32 amino acids beyond K22. The *ura4 -294* mutation affects a highly conserved residue (Ura4-D227N) in a highly conserved region of the protein.

## Description

The *ura4* gene of fission yeast encodes a 264 amino acid long orotidine 5'-phosphate decarboxylase protein that is broadly and strongly conserved across taxa (Grimm *et al.* 1988; Wood *et al.* 2012). This enzyme catalyzes a key step in pyrimidine biosynthesis; *ura4* mutants are unable to produce uracil de novo, but grow well if uracil is provided in the media. Reciprocally, if cells are provided with a substrate analog called 5-fluoroorotic acid (FOA), cells that express a functional Ura4 protein convert FOA to highly toxic 5-fluorourocil and are killed. Thus, the *ura4* gene supports both positive selection (only *ura4* wild-type cells will grow on media that lacks uracil) and negative selection (only *ura4* mutants will grow on media that contains FOA). We sought to adapt this system to explore mechanisms of meiotic recombination and we reasoned that we could take advantage of previously defined alleles, *ura4-595* and *ura4-294* (Fox *et al.* 1997). We therefore obtained strains harboring those alleles from the authors and, for the sake of independent confirmation, from an additional laboratory. As expected, haploid cells with the *ura4-595* and *ura4-294* alleles were auxotrophic for uracil and resistant to FOA ( **Figure 1B** ). However, the DNA sequences that we obtained—and which we confirmed by sequencing both strands of each allele and by sequencing alleles from different laboratories—differed from those reported previously.

The *ura4-595* allele purportedly had a duplication of GATC at bp position 595 (Fox *et al.* 1997). However, there is no GATC in that position within wild-type *ura4* . Moreover, our analyses revealed that the *ura4-595* allele actually harbors a four bp duplication of bp +63 to +66 (5’-CAAG-3’) within the ORF (these coordinates are numbered relative to the first nucleotide of the start codon) ( **Figure 1C** and **1D** ). This type of mutation is most consistent with a template slipping mechanism during DNA replication, whereby the DNA polymerase loses its register on the template strand, backs up a short way, then resumes elongation from the new register. The four bp duplication leads to a frameshift for translation, resulting in a truncated protein whose sequence is scrambled for 32 amino acids beyond the lysine at residue 22 ( **Figure 1E** ). Correspondingly, the mutant cells lack a functional Ura4 protein and are auxotrophic for uracil ( **Figure 1B** ).

The *ura4-294* allele purportedly had a C to T mutation at position 1212 (Fox *et al.* 1997). However, our analyses revealed that the *ura4-294* allele actually contains a G to A mutation at position +679 ( **Figure 1C** and **1D** ). This type of mutation is most consistent with incorporation of the wrong nucleotide by the DNA polymerase, either directly or indirectly following spontaneous deamination of the corresponding cytosine base in the complementary DNA strand. Either way, this change leads to a single amino acid substitution in the encoded protein (Ura4-D227N) ( **Figure 1E** ). This change, which is localized to a highly conserved residue within a highly conserved region of the protein, is sufficient to inactivate the protein and render the cells auxotrophic for uracil ( **Figure 1B** ).

## Methods

Strains of the indicated genotypes were constructed and propagated using standard fission yeast methods. Genomic DNA samples were prepared using smash and grab method with cells from 5 ml of culture. PCR and DNA sequencing were conducted using the listed oligonucleotide primers.

## Reagents

**Table:** 

Oligonucleotides:	
**Name**	**Sequence**
Ura4 FOR	5’- CCATCCCAGTTTAACTATGCTTCGTC-3’
Ura4 REV	5’- CGCCTAGGAAAACAAACGCAAACAA-3’

**Table:** 

Fission yeast strains:		
**Name**	**Genotype**	**Source**
WSP 0142	* h ^-^ ura4-294 *	Smith strain GP31
WSP 0263	* h ^+^ ura4-595 leu1-32 *	Smith strain GP191
WSP 0533	* h ^-^ ura4-294 *	Gould strain KGY145
WSP 0556	* h ^-^ ura4-D18 *	Gould strain KGY600
WSP 3776	* h ^-^ ura4 ^+^*	This study
WSP 8537	* h ^-^ ura4-595 *	This study
